# Digital Illness Narratives of Young Chinese Adults With Diabetes on RedNote: Qualitative Narrative Analysis

**DOI:** 10.2196/96543

**Published:** 2026-06-24

**Authors:** Zikun Liu, Donghan Fu, Yingjie Liu, Ying Meng

**Affiliations:** 1 School of Journalism and Communication, Wuhan University, 229 Bayi Road, Wuhan, 430072, China, 86 027 68752111; 2 School of Information Science and Learning Technologies, University of Missouri-Columbia, Columbia, MO, United States; 3 School of Media Science, Northeast Normal University, Changchun, China

**Keywords:** chronic illness, diabetes, social media, young people, illness narratives

## Abstract

**Background:**

Chronic illness disrupts everyday routines, social roles, and sense of self, particularly among young individuals undergoing identity formation. With the expansion of digital media, social platforms have become key sites where patients narrate illness experiences, negotiate stigma, and seek support. However, such processes remain underexplored in non-Western, collectivist contexts.

**Objective:**

This study examines how young Chinese individuals with diabetes construct illness narratives and negotiate identity in digital environments.

**Methods:**

This study uses a narrative analysis approach, combining inductive thematic coding with culturally and critically informed interpretation. A total of 303 narrative posts were collected from RedNote, a Chinese social media platform characterized by diary-like user-generated content. The dataset includes both text-based and video-based posts, capturing longitudinal and first-person accounts of living with diabetes.

**Results:**

In total, 4 distinct narrative types were identified. The chaos narrative captures experiences of cognitive dissonance, emotional breakdown, and disruption of daily routines following diagnosis, often accompanied by guilt toward family members and anxiety over future uncertainty. The stigma narrative reflects social withdrawal, concealment of illness, and perceived discrimination in intimate relationships and employment contexts, highlighting the role of externally imposed social judgment. The resilience narrative illustrates processes of self-acceptance, disciplined self-management, and the integration of illness into everyday life through routinized practices such as blood glucose monitoring and dietary regulation. The solidarity narrative emphasizes the importance of familial care and digitally mediated peer support, where users exchange practical knowledge, emotional encouragement, and collective identity markers, transforming isolation into shared experience. Across these narratives, illness is not only experienced as disruption but also actively reinterpreted through culturally embedded values such as familial responsibility and collective belonging.

**Conclusions:**

This study advances illness narrative research by demonstrating how digital platforms mediate culturally specific forms of meaning-making among young patients with chronic illness. It extends the concept of biographical disruption by conceptualizing it as a dynamic and relational process shaped by digital storytelling, familial expectations, and peer interaction. The findings highlight the importance of culturally sensitive and platform-aware approaches to health communication and digital patient support.

## Introduction

As one of the most prevalent chronic diseases globally, diabetes has increasingly eroded public health in China in recent years [1]. According to data from the International Diabetes Federation, the number of adult patients with diabetes in China continues to rise, reaching approximately 141 million by 2021, a 21.55% increase from that in 2019 [2]. Of particular concern is the growing impact of diabetes on young populations, as many struggle to manage their condition effectively despite rigorous treatment [3]. An epidemiological survey of diabetes in China revealed a prevalence of 2% among individuals aged 18‐29 years, with 20.2% of this cohort classified as prediabetic [4].

Often termed a “silent killer,” diabetes exhibits low cure and control rates, with prolonged illness risking irreversible tissue and organ damage, multiple complications, and lifelong dependence on medication or surgical interventions [5]. Protracted, unpredictable treatment processes frequently trigger anxiety, self-doubt, and depression, leading to high rates of diabetes-depression comorbidity that profoundly degrades the quality of life for patients and families [6]. For young individuals, traditionally idealized as the “rising sun” and “family’s hope,” diabetes interrupts not only personal aspirations but also familial expectations, imposing immense psychological and socioeconomic burdens when their health trajectories deviate from social norms [7]. However, the pervasive perception of diabetes as an “age-related disease” has led to systemic neglect in young patients [8].

Recent advancements in digital media have amplified the visibility of this marginalized group, enabling young patients with diabetes to document their illness journeys, share coping strategies, and forge peer support networks [9,10]. Through multidimensional narratives, young adults articulate the profound pressures of daily life, navigate emotional turmoil, and collaboratively explore pathways to self-healing and identity reconstruction [11]. As a result, digital narratives have emerged as key components of online health discourse, and scholars have examined how individuals share stories of health, illness, and caregiving across diverse digital platforms [11,12]. Patient-authored narratives provide first-hand insights into the lived experience of illness, capturing both the trauma of the disease and the evolution of self-identity [13]. Specifically, a recent study demonstrates that parents of children with diabetes in China experience identity disruption and family strain while using online narratives as culturally situated resources for reconstructing meaning under Confucian-Taoist sociocultural constraints [6]. Young adults with diabetes negotiated their biographical disruption by adopting shifting subject positions—concealing illness in public to preserve normality, asserting autonomy against medical control, and eventually developing a pragmatic acceptance that rendered diabetes a manageable part of everyday life [14].

More broadly, illness encompasses multidimensional experiences spanning the physiological, psychological, and social domains that evolve dynamically across life stages [15]. Within this framework, the concept of biographical disruption suggests that chronic illness destabilizes taken-for-granted assumptions about the self and the future, forcing individuals to reinterpret their identities and life trajectories [16]. This theoretical perspective highlights the importance of examining how these experiences are organized into narrative forms. However, existing research has not sufficiently examined how young adults themselves construct illness narratives in digital environments, nor how such narratives take shape as distinct forms of meaning-making. Addressing this gap requires closer attention to the narrative patterns through which young patients articulate their experiences and coping strategies. To address this gap, this study asks: What distinct types of illness narratives emerge from the experiences of young adults with diabetes? How do these narratives reflect the primary challenges faced by young patients and their coping strategies?

## Methods

### Study Design

This study uses a narrative analysis approach, combining inductive thematic coding with a culturally and critically informed interpretive framework to examine how illness narratives are constructed and negotiated in a digital context [17]. Rather than treating narratives as neutral reflections of experience, this approach understands them as culturally embedded meaning-making practices shaped by social norms, technological affordances, and broader discursive structures [18]. Initial coding was conducted following principles of reflexive thematic analysis as outlined by Braun and Clarke [19,20], while subsequent stages of analysis drew on illness narrative theory, particularly Frank’s typology, as well as sociological perspectives on stigma, identity, and digital culture [21,22]. In terms of illness narrative types, Frank classified patient illness narratives into 3 categories: restitution, chaos, and quest [23]. This dual approach enabled both data-driven pattern identification and theoretically grounded interpretation.

### Data Source and Sampling Strategy

Data were collected from RedNote, a Chinese social media platform characterized by user-generated lifestyle and health-related content [24]. The platform provides a space for young users to share personal experiences through text, images, and short videos, often in diary-like formats [3].

Given the exploratory and interpretive nature of this study, a purposive sampling strategy was used. Accounts were identified through iterative searching and long-term observation between March 2023 and April 2024, using a combination of diabetes-related keywords (eg, “diabetes,” “insulin,” and “blood sugar”). The sampling process prioritized accounts that met the following criteria: (1) self-identified young individuals (primarily university students) living with diabetes; (2) regularly posted first-person narratives related to illness experiences; (3) provided sufficiently detailed and longitudinal content for narrative analysis; and (4) represented noncommercial, nonpromotional personal accounts. Through this process, 13 accounts were selected as information-rich cases suitable for in-depth qualitative analysis. While the sample is not statistically representative, it is appropriate for narrative inquiry aimed at understanding meaning-making processes rather than population-level generalization. The final dataset comprised 303 narrative posts generated by 13 young adults living with diabetes, including 213 text-based posts and 90 video posts (Table 1). For video content, spoken narratives, captions, and on-screen text were transcribed verbatim, and relevant contextual cues (eg, tone, setting, and embodied expression) were documented to support interpretation.

**Table 1.** Detailed information of the selected 13 accounts.

	Sex	Followers, n	Posts	
			Text, n	Video, n
A1	Female	822	11	4
A2	Female	138	19	0
A3	Female	180	72	16
A4	Female	666	23	0
A5	Female	837	4	9
A6	Female	17	2	0
A7	Female	443	5	10
A8	Female	1661	12	11
A9	Female	209	38	3
A10	Female	583	2	9
A11	Male	713	2	12
A12	Female	9793	0	16
A13	Female	334	23	0

### Data Analysis

The analytical process proceeded in 3 iterative stages, and all authors participated in the coding process [19]. First, inductive coding: all posts were read multiple times to achieve familiarization. Open coding was conducted by the research team (ZL, DF, and YL) to identify recurring patterns related to illness experience, emotional responses, social interactions, and self-management practices. Coding was primarily conducted in Chinese to preserve linguistic and cultural nuances. Second, thematic organization: initial codes were grouped into broader thematic categories through iterative comparison and discussion among the researchers (ZL, DF, and YL). At this stage, the analysis remained largely inductive, focusing on patterns emerging from the data rather than imposing predefined categories. Third, narrative interpretation and theoretical integration: in later stages, the analysis moved beyond descriptive themes toward interpretive narrative analysis. Drawing on the illness narrative typology of Frank [23], as well as sociological theories of stigma, identity, and digital culture, the research team (ZL and DF) examined how different narrative patterns functioned as culturally situated meaning-making strategies.

Through this process, 4 narrative types were identified: chaos, stigma, resilience, and solidarity. While partially informed by existing theoretical frameworks, these categories were refined inductively to reflect culturally specific experiences in the Chinese context. Key analytical concepts, such as familial responsibility and filial piety, emerged through iterative engagement between data and theory. Discrepancies in interpretation were addressed through collaborative discussion among the authors until analytical consensus was reached.

### Reflexivity and Rigor

This study follows a reflexive approach to qualitative rigor, emphasizing transparency and interpretive awareness rather than positivist criteria such as intercoder reliability [25]. All authors are native Chinese speakers with cultural familiarity with the context under study, which facilitated nuanced interpretation but also required critical reflexivity. Reflexive journaling was maintained throughout the research process to document analytical decisions, emerging assumptions, and potential biases. These reflections informed ongoing interpretation rather than serving as a post hoc validation strategy. To enhance methodological rigor, the study engaged with criteria commonly used in qualitative research. Credibility was strengthened through repeated reading and iterative analysis of 303 narrative posts on RedNote, including both textual and video-based content, which enabled the refinement of emerging narrative patterns. Dependability was supported by documenting coding procedures, category development, and interpretive discussions throughout the analytical process. Confirmability was enhanced through reflexive discussions among the research team regarding how cultural familiarity and theoretical perspectives shaped interpretation, ensuring that findings remained grounded in the data. Transferability was addressed by providing contextual descriptions of the narratives, the experiences of young Chinese adults with diabetes, and the platform environment of RedNote.

### Translation and Positionality

All analyses were conducted in Chinese to preserve the integrity of the original narratives. Selected excerpts were translated into English by the first author (ZL) and independently reviewed by a bilingual researcher (YM) to ensure conceptual equivalence. Discrepancies were resolved through discussion. The research team’s shared linguistic and cultural background provided an insider perspective, which was both an analytical strength and a potential source of bias. Reflexive practices were therefore integral to the interpretive process.

### Ethical Considerations

This study analyzed publicly available social media content and did not involve direct interaction with users. In accordance with institutional guidelines for internet research, formal ethics board approval was not required [26]. However, given the sensitive nature of health-related narratives, several measures were taken to minimize potential harm following procedures adopted in previous research [27]. All user accounts were anonymized (A1-A13), and identifying details were removed or modified. Data were reported in aggregate form where possible, and excerpts were selected to avoid traceability through search engines. The study adhered to the terms of service of the RedNote platform and followed established ethical guidelines for research using publicly accessible online data. There were no human participants in this study, and informed consent was not required.

## Results

### Overview

We identified 4 distinct illness narratives among young adults with diabetes: chaos, stigma, resilience, and solidarity. While chaos and stigma narratives illustrate how these patients navigate a complex biopsychosocial landscape in which chronic illness disrupts identity, autonomy, and social integration, resilience and solidarity narratives highlight how individuals actively develop coping strategies, reframe meaning, and build support systems through both familial and networked relationships.

### Chaos Narrative

#### Overview

Many young patients with diabetes experienced significant cognitive dissonance, as their prediagnosis self-perception is disrupted by the sudden transformation into the identity of a “patient.” The chaos narrative reflected the biographical disruption, which was characterized by the upheaval of daily routines and a profound sense of existential uncertainty.

#### Cognitive Dissonance

Young patients frequently experience distress due to abrupt fluctuations in blood sugar levels compounded by disruptions in self-awareness. Due to their limited specialized knowledge of diabetes management, they often reported feelings of disorientation and helplessness. For instance, one young student recalled:

Being diagnosed with diabetes was a key turning point in my life. I kept asking myself, “Why did I get this disease?”

[A1, November 17, 2023]

During this process, they encountered confusion regarding their self-identity and discomfort with the social role transition. As one young student stated:

Sometimes I wonder, what is a “normal person”? Someone who can eat cakes on their birthday? Someone who does not require medication Someone who does not get sick or someone who is physically and mentally healthy?

[A4, September 7, 2023]

This transition from being perceived as “normal” to being labeled as “sick” generated emotional distress, as it disrupted deeply ingrained assumptions about selfhood and autonomy [28].

Within the Chinese cultural framework, where familial bonds and collective identity are paramount [29], diagnosis intensifies feelings of guilt and responsibility. Patients felt that the most direct effect was that their previously orderly and stable family life was abruptly thrown into chaos and disarray. As one patient remarked:

I lie in my hospital bed, staring at the ceiling, often crying secretly. I hate myself for being so useless, for getting sick, for making my parents suffer so much.

[A12, February 15, 2023]

Traditional Chinese ideals of filial piety deepen this sense of guilt, positioning young adults as responsible for economic success, parental support, and the continuation of family lineage. As a result, illness is moralized, becoming not merely a health condition but a perceived failure to meet culturally embedded expectations of responsibility and reciprocity.

Meanwhile, they were particularly concerned about the specific costs of hospitalization, and this keen awareness of financial pressures reflected their inner kindness and sense of responsibility. For example, a young female patient guiltily expressed:

I’ve been in the hospital for four days, and the cost has already reached 6,000. I really feel like I am a burden on my family. So, I pray that my condition is not serious, and that I can reduce the burden on my family. Please!

[A8, March 12, 2023]

These young adults, who have traditionally served as the central figures in the functioning of the family, influence internal interactions and relationships within the family. They also expressed an urgent desire to become independent in the future to alleviate the pressure on their families. For instance, as a young adult stated:

What makes me even guiltier is my parents. They have to spend a lot of money every month on insulin, medications, and various supplies. I just hope to graduate from university quickly and find a job so I can make money and reduce the burden on my family.

[A7, March 12, 2023]

This cultural paradigm significantly mediates how young Chinese patients construct the meaning of diabetes, as their identity and health management are inextricably linked to perceptions of familial duty and collective well-being.

#### Disordered Life

Chronic pain, endless medical treatments, loss of bodily functions, and altered physical appearance, which distinguish these young adults from others, significantly drain both the mental vitality and physical energy of the patient. For example, a young patient vividly depicted the embarrassment and inconvenience caused by the disease:

It’s so annoying, being woken up by hypoglycemia again. Now I have to sneak around like a midnight scavenger mouse, getting up quietly to eat without anyone knowing.

[A12, October 17, 2023]

Another patient shared a similar experience:

What does it feel like to be forced awake at 4 a.m? Low blood pressure, panic, no direction, rummaging through cabinets looking for anything to eat.

[A10, October 8, 2023]

These expressions deeply depicted the urgent responses and physiological states of patients with diabetes during nocturnal hypoglycemia, showing how they are physically bound by blood sugar fluctuations and psychologically bound by collapsing defenses.

The daily lives of these patients are disrupted by the arrival of the disease, leaving them with an overwhelming sense of uncertainty and unpredictability. For instance, as A4 (August 22, 2023) remarked, “When I was outside with hypoglycemia and my feet felt weak, I found that my glucose tablets had just run out; woke up in the middle of the night with hypoglycemia, and the nearest food was ten meters away.” Such experiences are common among patients. Like another young female patient: “Whenever I leave the house, my bag is always packed to the brim, with a blood glucose meter, an entire box of test strips, alcohol swabs, needles, and all kinds of snacks to raise my blood sugar, along with my newly refilled medication” (A2, January 2, 2024). The metaphor of a “heavy bag” poignantly symbolizes the relentless burden, stress, and anxiety associated with diabetes management, a burden that resonates with the insights of Brashers [30] into the emotional costs of chronic illness.

Furthermore, what should have been a life centered around learning and personal growth was transformed into one focused on illness and treatment. The disruption of academic pursuits, which were foundational for future development and life, shattered young adults’ hopes for the future. One patient expressed a sense of powerlessness:

Sudden hypoglycemia before the exam, who understands this sense of powerlessness? If I were already at the exam venue and had not brought candy with me, I cannot even imagine the consequences.

[A7, December 27, 2023]

This stress was further intensified by the sociocultural pressures inherent in the Chinese context, where the one-child policy places weight on an entire family’s expectations of a single child. One young female university student complained:

7.9 before bed, and when I open my eyes it’s 20.6. It’s terrifying! I am preparing for the exam. I am dying of exhaustion.

[A3, December 22, 2023]

For Chinese young adults, academic success is not merely a personal achievement, but a fulfillment of intergenerational obligations. Thus, the disruption of their educational trajectory not only jeopardized their future prospects but also exacerbated the psychological distress associated with chronic illness, as they struggled to balance both their health and the high social expectations placed upon them.

### Stigma Narrative

#### Overview

Meanwhile, the diagnosis of diabetes has prompted social withdrawal among young patients, exposing their experiences of stigma in daily life. The stigma narrative, which highlighted widespread misconceptions regarding insulin use and disease etiology, further reinforces structural stigma, thereby limiting patients’ opportunities and eroding their self-worth. Many patients concealed their conditions to avoid imposing perceived burdens on family dynamics or facing social exclusion.

#### Social Withdrawal

Diabetes has a profound impact on the lives of young patients, manifesting significant emotional fluctuations and altering their social dynamics. Many young patients with diabetes feared being labeled and worried about the judgmental stares of others. For example, a young female patient expressed:

I started to reject the invitation from guys around me, proudly calling myself a “single lady.” I was always afraid that others would find out about my “diabetes,” and even afraid of their odd stares.

[A3, November 30, 2023]

Likewise, as a young adult remarked:

I planned to pursue him, but in the second semester, I discovered my condition. I let him go with a fracture in my soul, now love lies beyond my reach.

[A8, April 2, 2023]

These expressions illustrate how the disease undermines the foundations of social interaction, an essential aspect of maintaining a coherent life course [31].

Social alienation and exclusion led young adults to actively withdraw from social activities and passively become excluded. Some patients were met with unfair treatment, which led them to adopt a strategy of “hiding their diabetes and refraining from disclosing their condition to others.” For example, as a young patient expressed:

What I did not expect was that my friends hearing about my condition did not even express any concern. They even dissolved the shared travel funds. I was sad and stunned. Maybe what I thought were good friends were just my own wishful thinking.

[A4, May 7, 2023]

This isolation aligns with previous research on the role of social context in shaping illness narratives [32], highlighting how misunderstanding and exclusion contribute to a sense of disillusionment regarding social relationships.

Simultaneously, due to the strict requirements of blood sugar control, many patients had to give up the activities they once enjoyed. One young female patient expressed her deep sense of loss and frustration by saying:

My hatred for diabetes reached its peak. When I see people eating well on the street, I feel sad. Just thinking about how it would make my blood sugar spike makes me angry.

[A12, June 7, 2023]

These narratives revealed that patients with suppressed pain experienced in managing their lifestyle and emotions as well as the internal struggles they endure while coping with the disorder in their lives. They are compelled to continually adapt to life with the illness while striving to maintain balance amid persistent challenges.

#### Social Discrimination

Many young patients expressed that the social “gaze” often made them ashamed of their identity as diabetics, and they were constantly seeking a balance between disease management and social expectations. For example, a university student shared how diabetes impacted her job opportunities by saying:

I did not get the offer because I have diabetes. Of course, there are so many “normal” people out there that they can be hired. I can only comfort myself that every boss wants to hire “normal” people.

[A6, July 31, 2023]

Although patients may not directly encounter overt criticism, they perceive stigma through nonverbal reactions, social and cultural responses, media portrayals, and their own imagination. Likewise, one young female patient shared: “I used to think I was different from normal people, hiding in school to inject insulin secretly” (A10, November 9, 2023). Such experiences illustrate that stigma is transmitted not only through overt social interactions but also through subtle, nonverbal cues and media portrayals, ultimately coercing patients to adopt unsustainable adaptations in managing their disease.

Moreover, reliance on insulin is frequently misinterpreted as ostentatious behavior or a marker of indulgence. As one patient remarked:

Some say we are showing off when we inject, others say we eat too much sugar, and some even use us as a cautionary tale. There’s nothing we can do.

[A12, January 20, 2024]

The stigma caused by the physical damage inflicted by diabetes is transformed into a process of psychological distress. For them, the pain of the treatment process was not only the discomfort of diabetes itself and the side effects of treatment but also the sense of loss from being misunderstood. For example, one patient stated:

I hardly ever consumed sugary drinks. I can only say that I had type 1 diabetes by chance, by something unknown. Please give us more attention and less misunderstanding.

[A11, February 3, 2024]

These appeals reflected the patients’ desire to raise awareness of diabetes and reduce discrimination.

### Resilience Narrative

#### Overview

Many patients demonstrated remarkable emotional resilience, evident in their self-acceptance, gratitude, and proactive reframing of their disease experiences. By developing structured, yet flexible self-care routines, they reclaimed agency over their health and identities. Thus, resilience narratives constitute a pivotal element of coping strategies.

#### Emotional Fortitude

Young patients with diabetes often undergo psychological transformation and self-acceptance when facing the disease. They adapted to living with diabetes by actively recording their blood sugar levels. For instance, as a young patient shared:

I have been studying increasingly harder. I have maintained records for about two months. Although I cannot fully accept this disease yet, looking back, it feels like the boat has already sailed through a thousand mountains.

[A13, September 19, 2023]

Patients exhibited a positive attitude and self-affirmation when facing the challenges of the disease, and they had a clear understanding of and plan for managing their condition. As a patient stated:

I actively learn about blood sugar control, study the glycemic index of different foods, and calculate carbohydrate ratios, and continually try new methods. I love this version of myself I will always be proud of who I am! We can be sugar warrios.

[A3, December 7, 2023]

Such narratives resonated with the concept of narrative reconstruction [13], where the integration of daily health regimens into one’s life story facilitated a shift from seeing diabetes solely as a source of illness-related burden to recognizing it as a catalyst for personal growth.

In addition, they expressed gratitude for their lives. Coping with the disruption of their life course and using surrounding resources often involves altering their self-awareness and rethinking the direction of their lives. Some patients expressed their gratitude for the availability of insulin, which enabled them to live a normal life and enjoy life’s pleasures. As a patient remarked:

I’ll be taking my first insulin shot on my 20th birthday, marking the 504th day of living with diabetes. Thanks to insulin, I can now enjoy this sweetness like a normal person.

[A8, April 1, 2024]

Other patients compared insulin to their “crystal energy,” saying: “I eat in two steps: the first step is to check my blood sugar, the second step is to inject my crystal energy to stabilize my condition” (A11, March 15, 2024). Within this disarray, patients engaged in narrative reinvention and acts of symbolic reclamation that transformed vulnerability into agency. These positive attitudes and the desire for a healthy lifestyle demonstrated their optimism and discipline in managing their disease. In the context of disruption caused by past experiences and anticipated futures, they reconstructed their present experiences by adopting a proactive and positive approach to life.

#### Self-Routinization

Many patients demonstrated how they normalized the blood sugar management process. The philosophy of living in the present moment serves as a narrative for patients to reconstruct their lives after diabetes, aiding them in facing life after diabetes. For example, a female university student shared:

I always separate dry and wet food when eating. Foods that are too mushy are not suitable for blood sugar. Today, it’s still a day full of energy!

[A3, June 9, 2023]

In line with ancient Chinese traditions, the notion of living in the present moment offered hope for a turning point even amid severe disruptions, suggesting that although diabetes may significantly disrupt life, hope remains, and one must confront present difficulties with a balanced mindset.

In addition, some patients integrated diabetes into their lifelong journeys. For example, one patient wrote:

I regularly monitor my blood sugar, and there have been no complications so far. Diabetes has kept me disciplined for life.

[A7, January 12, 2024]

They recognized that balancing relaxation and enjoyment of life is an essential part of maintaining health management. Similarly, a female university student reflected:

I feel much better because I know that blood sugar control is a lifelong journey, and allowing me to relax a little helps me to continue. I believe I will become stronger and stronger in managing my blood sugar.

[A10, October 21, 2023]

The perseverance and adaptability they displayed in managing their disease reflected their determination and courage in pursuing personal passions and fulfilling life needs.

Moreover, travel adventures have become a common means of solace for young patients with diabetes. As one shared:

This insulin shot is from snowcapped mountains. Disease is not a stumbling block on the road to fulfilling my dreams.

[A12, December 10, 2023]

During these journeys, they enjoyed exploration with a positive attitude while carefully managing their blood sugar, embracing every new adventure with health and resilience. Another patient shared a similar travel experience by saying:

Nanchang is a great city! I walked over 20,000 steps per day, and my blood sugar remained stable.

[A1, January 7, 2024]

### Solidarity Narrative

#### Overview

The patients highlighted that kinship and digital social support are pivotal in enabling young patients with diabetes to adapt to chronic illnesses. These support systems foster resilience by strengthening familial bonds and enhancing engagement within digital communities. The narrative of solidarity illuminated familial care, and online networks provided both instrumental guidance and empathetic support, effectively transforming isolation into collective empowerment. By leveraging shared knowledge and reciprocating support, patients not only navigate disease management but also cultivate a robust culture of mutual aid.

#### Familial Solidarity

Although diabetes disrupted these young adults’ life trajectories and strained their families, it also fostered resilience and strengthened their familial bonds. Most patients emphasized the meticulous care provided by their families throughout their disease journey. For instance, one patient stated:

I measure my blood sugar every two hours at night, and my mother and sister have been taking turns staying with me. I feel so blessed to have my family with me.

[A11, February 15, 2024]

Notably, many young patients with diabetes specifically mentioned the care provided by their fathers after diagnosis. In traditional Chinese culture, a father’s love is often expressed through authority and strength. However, many fathers chose to express their affection through tenderness and care when confronted with their children’s illnesses. For instance, one patient recalled:

When we reached the store, I pointed to the cream puff and told my dad it was delicious. He asked the clerk, “Does this contain sugar?” At that time, I began. My dad always encouraged me to try any food that I wanted to. In the past, I would get very upset, but now I understand this is another form of love from my father.

[A10, January 1, 2023]

The father’s actions deeply illustrated unspoken love, encouraging his child to live courageously, and the child, in turn, gradually felt and appreciated this profound paternal affection, learning to accept and value support from their parents in managing the disease. Similarly, a patient expressed that the disease provided an opportunity to rebuild their familial bond:

After I got diabetes, I felt like the conversations between my dad and I became much more frequent. He asks me every day what I want to eat, what dish I’d like for dinner, and what fruits I’d like for tomorrow.

[A11, January 9, 2024]

In facing the destruction caused by illness, most patients mentioned the significant role that family care and encouragement played at different stages of their illness. Even though family relationships may become disordered or chaotic after the onset of illness, their unwavering support gradually helped to mitigate the relational fractures caused by the disease.

#### Reciprocal Digital Community

The mutual understanding and support among those with diabetes online helped them better manage the disease and served as an important pillar in their lives. As natives of the digital age, young patients with diabetes actively used social platforms to cope with challenges, sharing their daily experiences with online diabetes friends to seek emotional support and a sense of belonging. One patient wrote:

I am truly thankful to everyone here, for making me no longer feel like an island. As fellow members chose diabetes, we really feel like a family on the Internet.

[A4, December 27, 2023]

This finding echoes earlier research, showing that digital interactions can transform feelings of isolation into collective empowerment, highlighting how online health narratives both shape and are shaped by their social contexts [32]. When faced with day-to-day challenges such as preparing for a graduate school entrance interview, patients turned to these virtual networks for guidance. As a patient remarked:

I’m about to attend a graduate school entrance exam interview soon, which includes both a written test and an interview. I want to ask any experienced friends with diabetes. Can I wear an insulin pump during the interview?

[A3, March 23, 2024]

Online support from netizens continued to be a stable provider of ongoing social support for patients in instrumental and emotional support.

Through the process of identity switching and reinforcement, patients found new meanings in their relationship with the disease, including acceptance of life and disease as natural, a sense of gratitude and reciprocation, the pursuit of value realization and social recognition, and a life attitude of coexistence with the disease. For example:

Dear fellow diabetics, please don’t be sad. There are millions of people in the world who are like us. We can lift our heads and embrace ourselves in the most vibrant winds.

[A1, November 17, 2023]

Young patients with diabetes not only actively adjusted their mindset through self-encouragement but also inspired others who were in similar situations. Another girl shared the following tips:

I just want to share how I carry my little Susu (insulin) when I go out. I bought this storage bag from an online platform, and it was about the length of my arm. Let me show you the inside. It can hold two–three pens, and there is space for ice packs, alcohol swabs, needles, and other essentials. Everything fits perfectly inside.

[A12, October 12, 2023]

By sharing their practical life experiences, they actively helped others and showed a strong sense of responsibility and empathy. Some patients also provided detailed guidance on dietary considerations for diabetes management, helping others better control their blood sugar levels. For instance:

Going from rich, heavy-flavored meals to a relatively lighter, sugar-controlled diet is definitely hard to adjust to. My approach is trying to eat until I am 70% full, and use lighter cooking methods to taste the natural flavors of food.

[A5, March 20, 2024]

As they deepened their understanding of blood sugar control, many patients shared their sugar control experiences:

Match insulin well, and I can eat what normal people eat. Sugar-free or zero-calorie coffee has almost no effect on blood sugar, while thicker milk causes an increase of 1‐2 points.

[A13, October 10, 2023]

Young patients with diabetes offer both instrumental and emotional support to one another through online platforms. These findings align closely with the literature on digital health narratives and support networks, underscoring the critical role of online communities in chronic illness management [13].

## Discussion

### Principal Findings

This study examined how young Chinese individuals with diabetes construct illness narratives in digital environments. Through narrative analysis of 303 posts on RedNote, 4 narrative types were identified: chaos, stigma, resilience, and solidarity. These narratives demonstrate that chronic illness is experienced not only as a biographical disruption but also as an ongoing process of identity reconstruction shaped by cultural expectations, social pressures, and digital interaction.

### Cultural Context and Illness Narrative Reconstruction

The findings indicated that young patients with diabetes face intertwined dilemmas of biographical disruption and identity reconstruction, requiring them to renegotiate their lifestyles, social roles, and self-perceptions. In the Chinese cultural context, where individuals are embedded within interdependent familial structures, illness is often experienced not solely as a personal condition but as a disruption to familial continuity and social expectations [33]. Diabetes-induced guilt therefore intensifies identity crises, as patients may perceive their illness as a failure to fulfill filial responsibilities and future obligations [34]. These pressures are further reinforced by broader structural conditions, including the legacy of the one-child policy, competitive educational systems, and familial expectations surrounding academic achievement, economic success, and marriage.

Situating these findings within the Chinese cultural context helps explain the distinctive features of these narratives. This study offers a culturally grounded reinterpretation of the illness narrative framework of Frank [23]. In the Chinese context, chaos narratives are often intertwined with culturally specific elements such as familial guilt and moral responsibility, which extend the scope of the original concept. Regarding the other narrative types, they reflect contextually grounded variations and emergent categories shaped by the sociocultural and digital environment under study. For example, the resilience narrative differs from restitution, in that it does not center on returning to a preillness state, but rather on ongoing adaptation and disciplined self-management. The stigma narrative captures socially imposed processes of devaluation that are not fully encompassed by chaos, while the solidarity narrative highlights digitally mediated forms of collective coping that extend beyond the individual-oriented trajectory implied in quest narratives.

### Digital Solidarity and Platform-Mediated Identity Formation

At the same time, the findings reveal how digital environments reshape both illness identity and health governance. Young patients were simultaneously burdened by intergenerational pressures derived from Confucian family ethics while benefiting from forms of “liquid solidarity” fostered through online communities [35]. Through narrative sharing, peer interaction, and emotional support, patients actively engaged in self-healing, meaning-making, and identity reconstruction. Familial support, social networks, and digitally mediated peer communities emerged as critical resources that enabled patients to normalize illness experiences, reframe emotional distress, and gradually develop systems of self-care. Frequent self-referencing as “sugar warriors” reflected a shift in illness identity from stigma toward a digitally networked form of resistance and collective belonging. This transformation aligns with Castells’ [22] framework of identity formation in network society, in which patients construct counter-public spaces and solidarity communities through algorithmically connected peer networks.

### Health Neoliberalism and Digital Self-Management

However, these digitally mediated forms of empowerment also operate within broader structures of health neoliberalism. As bodily functions increasingly become tied to ideals of productivity, discipline, and self-optimization, chronic illness may be framed as a form of personal failure rather than a socially embedded condition. Within this context, seemingly neutral disease-related content on digital platforms often reproduces a “self-management supremacist” governance logic that transforms structural inequalities into matters of individual responsibility [36]. Consequently, digital illness narratives function not only as spaces of support and resistance [37] but also as sites where broader moral expectations surrounding health and self-discipline are reproduced and negotiated. This tension highlights the ambivalent role of digital platforms, which simultaneously enable collective empowerment and reproduce normative pressures surrounding health, productivity, and self-governance.

### Clinical Implications

Rather than focusing solely on individual-level coping or behavioral change, the findings of this study point to the need for a structurally informed approach to diabetes care and communication. The narratives analyzed here reveal how responsibility for disease management is often moralized and individualized, particularly within digital environments shaped by health neoliberalism. As such, clinical and public health interventions should move beyond emphasizing self-discipline and adherence, and instead work to demoralize chronic illness by recognizing the structural, cultural, and psychosocial conditions that shape patients’ experiences.

At the clinical level, this calls for greater sensitivity to the moral and emotional burdens associated with self-management, particularly in contexts where familial expectations and intergenerational responsibility intensify patients’ sense of guilt. Clinicians should be attentive to how patients interpret illness in relation to identity, family roles, and social expectations. At the platform level, the findings suggest the need to critically examine how digital health content and algorithmic visibility may reinforce a “self-management–centered” logic. Social media platforms should consider how to diversify representations of illness experiences, making space for narratives that reflect structural constraints, ambivalence, and nonlinear coping trajectories, rather than privileging idealized forms of disciplined self-management. More broadly, this study calls for a shift in health communication from framing diabetes as an individual failure of control to understanding it as a socially and culturally mediated condition, shaped by intersecting forces of family structure, educational pressure, and digital media environments. Such a shift is essential for developing more equitable and context-sensitive approaches to chronic illness care.

### Limitations

This study has several limitations. First, the sample consists of 13 users from a single platform, which limits the generalizability of the findings. Although purposive sampling was used to identify information-rich cases, the dataset may not capture the full diversity of illness experiences among young people with diabetes. The predominance of female college students in the sample may also reflect platform-specific participation patterns rather than broader population characteristics. Second, the study focuses on publicly available social media content, which provides valuable insights into self-expression but may not fully represent offline experiences or less visible forms of coping. Users may selectively present certain aspects of their lives while omitting others, leading to potential bias in the narratives analyzed. Third, the platform context of RedNote may shape both the style and content of narratives. As a platform oriented toward lifestyle sharing and social engagement, it may privilege particular modes of storytelling while marginalizing others. Future research could compare multiple platforms to better understand how different digital environments influence illness narratives. Fourth, this study relies solely on publicly available user-generated content and does not include interviews with patients or family members. As a result, interpretations of family dynamics are based on patients’ self-representations. Future research could combine digital trace data with interviews to strengthen validity.

### Conclusions

This study demonstrates that illness narratives among young Chinese individuals with diabetes are shaped by the intersection of cultural values, social structures, and digital media environments. By identifying 4 narrative types, the study highlights how patients move beyond disruption to actively reconstruct identity, negotiate stigma, and build forms of resilience and solidarity. More broadly, the findings suggest that digital platforms are not merely spaces for sharing experiences, but active sites of meaning-making that transform how chronic illness is understood and managed. Recognizing these dynamics has important implications for health communication, particularly in designing culturally sensitive and platform-aware interventions that support young patients in navigating chronic illness. Rather than viewing diabetes solely as a biomedical condition, this study underscores the importance of understanding it as a socially and culturally mediated experience. Future research should further explore how digital storytelling shapes long-term health outcomes and how intergenerational communication may mitigate the moral pressures associated with illness in collectivist contexts.
